# Research hotspots and trends of plumbagin: A bibliometric perspective

**DOI:** 10.1097/MD.0000000000041726

**Published:** 2025-02-28

**Authors:** Zichen Xu, Yuqi Xin, Xinjian Zhang, Jianwei Liu, Yang Liu, Runying Guo, Qingkun Jiang, Jiaxuan Qiu

**Affiliations:** a Department of Stomatology, The First Affiliated Hospital of Nanchang University, Nanchang, Jiangxi, China; b Department of Stomatology, Jiangxi Provincial Key Laboratory of Oral Diseases, The First Affiliated Hospital, Jiangxi Medical College, Nanchang University, Nanchang, Jiangxi, China; c Medical College, Nanchang University, Nanchang, Jiangxi, China.

**Keywords:** plumbagin, bibliometrics, visualization

## Abstract

Plumbagin is a biologically active naphthoquinone compound. Research related to plumbagin has gained popularity, evidenced by a gradual increase in publications. However, a bibliometric study in this field has yet to be conducted. Consequently, this study aims to evaluate the global scientific output of plumbagin research through bibliometric analysis, explore the status of research in this field over the past 15 years, and predict its future research hotspots. Visual analysis software, including CiteSpace, VOSviewer, and the R package ‘bibliometrix’, was employed to analyze all literature pertaining to plumbagin published between 2009 and 2024. Bibliometric records were sourced from the Web of Science Core Collection. This study analyzed a total of 2061 publications. China was found to have the largest number of publications, while the Council of Scientific & Industrial Research recorded the highest publication count in this field. Thomas and David D were identified as the authors with the most publications. The journal with the highest number of citations was *J Biol Chem*, and Padhye S was noted as the author with the highest citation count. In recent years, cancer treatment has emerged as the most closely related research topic concerning plumbagin, and keyword cluster analysis highlighted ‘sarcoplasmic reticulum’ as a prominent research keyword. Through quantitative and visual analysis of plumbagin, this study reveals that plumbagin research remains a valuable field. Anticancer treatment of plumbagin is identified as a future research direction.

## 1. Introduction

Plumbagin (PLB) is a biologically active naphthoquinone compound. The earliest report on PLB was a paper published by scholar Hoskin in Biochim Biophys Acta in 1962. This article explored that when guinea pig brain slices were incubated with glucose, adding the same amount of PLB could slightly stimulate glucose metabolism and oxygen utilization. PLB has a wide range of pharmacological functions. Studies in vivo and in vitro have shown that it has antibacterial,^[1]^ antifungal,^[2]^ anti-atherosclerotic,^[3]^ and antitumor^[4]^ effects. At the same time, PLB has the characteristics of mild Chinese herbal medicine, and can be used in the adjuvant treatment of a variety of diseases.

The research direction of PLB in the past 2 decades mainly focuses on antitumor and anti-inflammatory aspects. In 1984, Wurm and other scholars found that PLB is the main material basis of PLB plant antitumor,^[5]^ which is the first report of PLB in the antitumor direction. With the growing significance of traditional Chinese herbal medicine in tumor treatment, there are an increasing number of reports on PLB. In 2003, Lin et al^[6]^ found that PLB can significantly inhibit the proliferation and growth of Calu-1, HeLa, Raji, and wish tumor cells. In the next 10 years, scholars began to gradually explore the specific mechanisms of PLB against cancer, including inhibiting the proliferation of melanoma cells through the c-Jun signaling pathway,^[7,8]^ killing non-small cell lung cancer through the ERK signaling pathway,^[9]^ inducing apoptosis through the p53 signaling pathway to inhibit the growth of osteosarcoma cells,^[10]^ inhibiting the proliferative activity of breast cancer and non-small cell lung cancer through the PI3K/Akt signaling pathway,^[11,12]^ killing pancreatic cancer cells through the EGFR and STAT3 signaling pathways,^[13]^ and acting on the NF-κB signaling pathway to inhibit the proliferation of breast and gastric cancer cells,^[14,15]^ and act on AMPK signaling pathway to kill colon cancer cells.^[16]^

The first report on the anti-inflammatory effect of PLB was in 2006 when Sandur et al^[17]^ found that PLB could inhibit inflammatory stimuli. In the following 10 years, Luo et al^[18]^ found that PLB could reduce the production of pro-inflammatory cytokines IL-1, IL-6, and TNF, as well as inhibit NF-κB activation exerting anti-inflammatory and analgesic effects. Wang and colleagues^[19]^ found that PLB inhibited NF-κB and MAPK signaling in RAW 264.7 cells stimulated by lipopolysaccharide to downregulate the expression of pro-inflammatory mediators, thereby exerting its anti-inflammatory effects. Zhang et al^[20]^ found that PLB protects Wistar rats from oxidative stress and inflammation induced by spinal cord injury by upregulating NRF-2. Zhang et al^[21]^ found that PLB can activate the AMPK pathway to reduce inflammation, oxidative stress, and obesity-related asthma.

In recent years, with the rise of nanomaterials research, the drug delivery platform of PLB is one of the research hotspots. Kumar et al^[22]^ found that PEGylated liposomes can provide a delivery platform for PLB, which has the effect of enhancing the plasma half-life, thus showing better antitumor efficacy. Qiao et al^[23]^ developed a pH-responsive bone-targeted drug delivery system loaded with PLB to treat bone metastasis of breast cancer.

The biological application of PLB has been discussed in many journals; however, these studies mainly focus on the current state of the overall biological application. Bibliometrics is an interdisciplinary science that quantitatively analyzes all knowledge carriers through mathematical and statistical methods. It is a comprehensive knowledge system integrating mathematics, statistics, and philology and pays attention to quantification. Bibliometrics mainly carries out quantitative and qualitative analysis on different indicators such as countries, institutional authors, and keywords of published journal data. It can be used to statistically evaluate the most influential countries/institutions and authors at present and can also predict future hotspots and trends through existing information^[24]^ At present, many biomedical applications of Chinese herbal medicine have been well studied and explored through bibliometric analysis. Chen and other scholars conducted a bibliometric analysis of the research status of Astragalus membranaceus and discussed the prospects and challenges of Astragalus membranaceus in biomedical research.^[25]^ Zhang and other scholars used VOSviewer and CiteSpace software tools to comprehensively summarize the research trends related to licorice in the past 25 years.^[26]^

This analytical approach is becoming critical for policy formulation and determining current research trends. However, so far, no study has objectively summarized or analyzed the research trend of PLB, especially from the perspective of bibliometrics. Therefore, this study uses bibliometric methods to conduct a macro review of the existing studies of PLB from 2009 to 2024 and predicts the possible research hotspots in the future.

## 2. Materials and methods

### 2.1. Search strategy and data collection

Data were obtained from the Web of Science (WOS) Core Collection from January 1, 2009, to March 1, 2024. A total of 2061 publications were obtained using a fine search method to accurately present the development trajectory and frontier research of PLB biological application. The search formula used in this study is “plumbagin” or “PLB.” This bibliometric analysis only includes original research articles and review articles. Literature that did not match the study and duplicates were manually excluded.

### 2.2. Bibliometric analysis

In this study, CiteSpace, VOSviewer, and the R package “bibliometrix” software were used to visualize and analyze the data. We imported the data obtained from the WOS Core database into CiteSpace, VOSviewer, and Bibliometrix for analysis. Firstly, bibliometric distribution is carried out according to the data of year, country, research institutions, authors, references, and keywords. Then, based on the analysis of the identified keywords, the research trends and hotspots of the biological application of PLB were further analyzed, providing a reliable direction for the long-term research and clinical transformation of PLB.

CiteSpace is a Java-based software developed by Professor Chaomei Chen for the study of scientometrics knowledge atlas.^[27]^ The software can guide the visualization of research hotspots and the evolution process in various fields based on existing literature and predict the development trend of various fields.^[28]^ This effective tool for large-scale information analysis can explore and visualize important events and trends in a given field. In recent years, it has become a key tool for bibliometric analysis. In this study, CiteSpace (version 6.2.r7) and Bibliometrix based on R language carried out bibliometric analysis.

VOSviewer was founded by the Science and Technology Research Center of Leiden University in 2010.^[29]^ VOSviewer, as software for creating and analyzing bibliometric networks, integrates literature information and visualizes authors, institutions, and keywords.

Bibliometrix is an open analysis tool developed by Massimo Aria and Corrado Cuccurullo for quantitative analysis of science and bibliometrics.^[30]^ It is simple to operate, avoids the tedious multi-step operation of the user on the software processing interface, and reduces the error probability at the same time. It is relatively easy to complete highly repetitive and multi-step computational tasks.

## 3. Results

### 3.1. Search results of related studies on plumbagin

**Figure 1.**
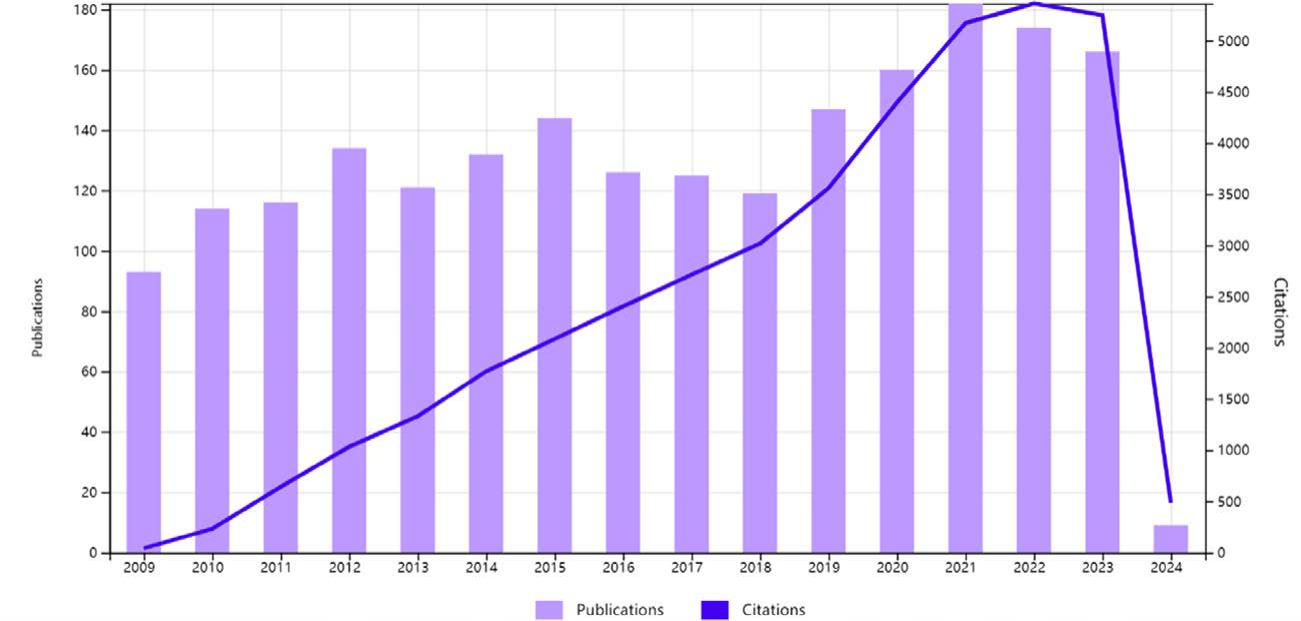
Number of publications and citations over the past 15 years.

The language of the articles included in this study is English, and the type of literature is paper and review. Based on the adopted search strategy, a total of 2061 articles were obtained from the WOS Core Collection. Over the past 15 years, the number of papers on the biological application of PLB has generally shown an upward trend. 2021 witnessed the highest number of papers published, with a total of 182 papers. Overall, these results indicate that the biological application of PLB has attracted extensive interest from medical researchers worldwide (Fig. 1).

### 3.2. Country/region analysis

**Figure 2.**
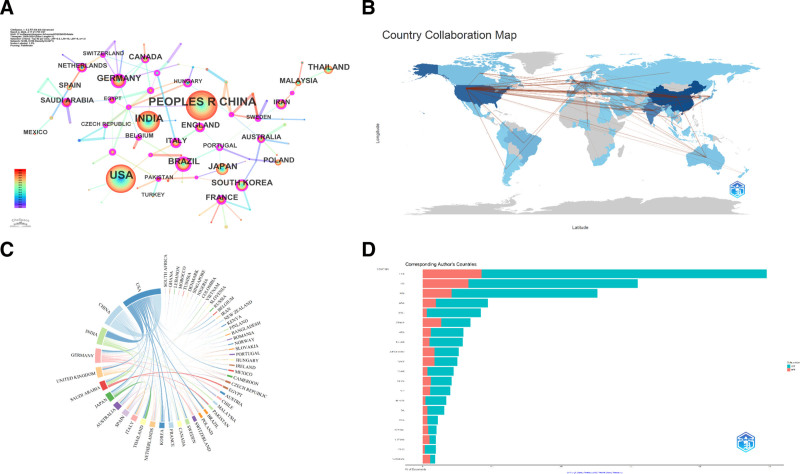
(A) National distribution of plumbagin studies. (B) Map of cooperation among countries. (C) Chord diagram of cooperation among countries. (D) Cooperation of the corresponding author’s country.

We utilized CiteSpace, Bibliometrix, and VOSviewer to analyze the latest level of articles on PLB in each country or region. The size of nodes indicates the number of files published in each country. The purple color on the outer ring of the node represents the high centrality of the country (centrality > 0.1), indicating greater international influence. The country/region distribution map consists of 86 nodes, indicating that 86 countries or regions have studied PLB. The top 5 countries with the largest number of documents are People’s Republic of China (n = 524), USA (n = 424), India (n = 286), Japan (n = 115), and Germany (n = 104; Fig. 2A).

The results show that although China has published a large number of papers on PLB, the number of cores is <0.1, indicating that China has not yet invested in forming its own international cooperation network. The United States is the country with the second largest number of publications, but the cores is also <0.1, indicating that the United States, like China, has not yet invested in forming its own international cooperation network, which may be caused by the inconsistent research directions of PLB between the 2 countries. The country with the highest number of centers is Portugal, indicating that Portugal has formed its own international cooperation network for the research of PLB. At the same time, we can see the close cooperation between the top 3 countries in terms of the number of papers published through the chord diagram (Fig. 2B–D).

### 3.3. Author, institution and discipline analysis

**Figure 3.**
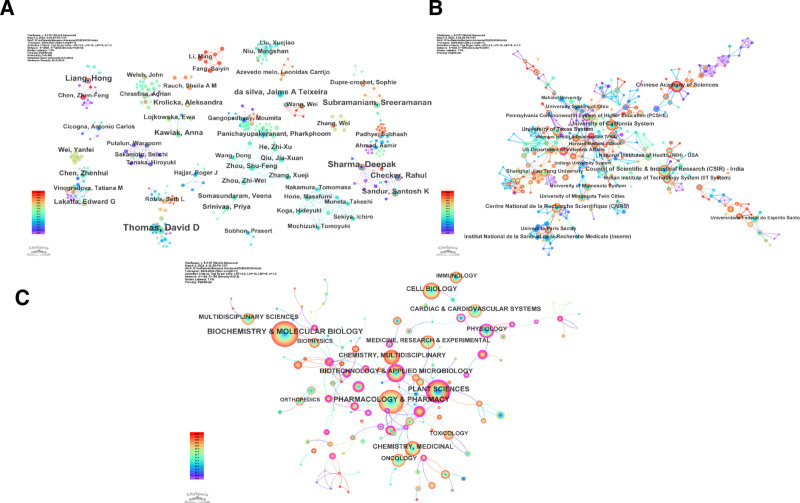
(A) Distribution of authors of plumbagin research. (B) Institutional distribution of plumbagin research. (C) Subject distribution of plumbagin research.

At the same time, we used CiteSpace and Bibliometrix to analyze the number of papers published by research authors and obtained 4563 nodes, indicating that 4563 scholars studied PLB. Among them, 4 scholars have published 10 or more articles on PLB. They are Thomas, David D (16), Sharma, Deepak (13), Liang, Hong (10), and Subramaniam, Sreeramanan (10). The research fields of these 4 authors are different, mainly involving biochemistry, oncology, and immunology (Fig. 3A). Among them, Thomas and David D’s research on PLB is mainly about its transport effect on ca^2+^ and plant science.

For the issuing institutions, we also used CiteSpace for analysis and obtained a total of 655 nodes, indicating that 655 institutions have studied PLB. Among them, 4 research institutions have published more than 30 articles, namely, Council of Scientific & Industrial Research–India (36), University of California system (33), Chinese Academy of Sciences (33), and Centre National de la Recherche Scientifique (30; Fig. 3B). Among them, Council of scientific & Industrial Research, India has extensive research on PLB, involving toxicology, antibacterial, angiogenesis and many other directions.

We used CiteSpace to analyze PLB discovery involving many disciplinary areas. The research on PLB mainly involves 3 disciplines: Biochemistry & Molecular Biology, Pharmacology & Pharmacology, and Plant Sciences. Based on the above results, it is evident that PLB is a Chinese herbal medicine with research value (Fig. 3C).

#### 3.3.1. Co-citation analysis

**Figure 4.**
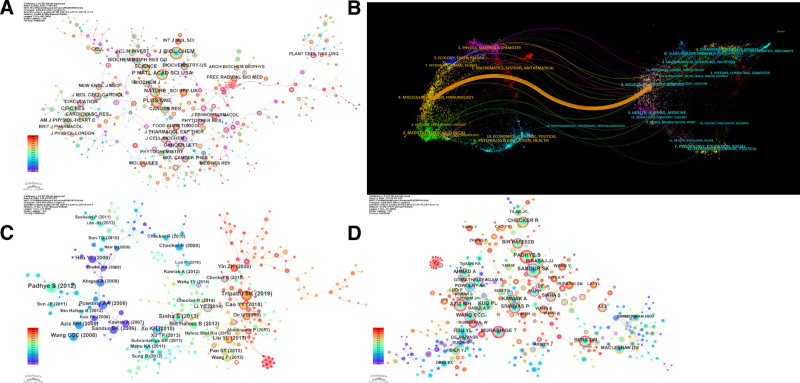
(A) Journal cocitation distribution of plumbagin research. (B) The double map coverage of plumbagin research was cited. (C) Literature distribution of plumbagin research. (D) Cocitation distribution of plumbagin research authors.

For the cocitation analysis of journals, we found that PLB appeared in 466 journals. The top 5 journals where PLB was cited the most include Journal of Biological Chemistry (746), P Natl Acad Sci USA (604), PLoS One (568), Nature (509), and Science (376; Fig. 4A).

To better understand the relationship between different research fields involved in the study of PLB, a dual-map coverage of journals was conducted to represent the distribution of cited and citing journals. The double-graph superposition identifies a major orange path, indicating that research published in the Journal of Molecular/Biology/Immunology is usually cited in research belonging to Molecular/Biology/Genetics (Fig. 4B).

For the cocitation analysis of literature, we found a total of 527 nodes, indicating that 527 articles on PLB were cited. Among them, the most cited article was published by Padhye in Med Res Rev in 2012, with an impact factor of 9.3. This article mainly summarizes the chemical properties, biological activities, and medicinal value of PLB. The articles cited more than 30 times are shown in the table below (Fig. 4C).

For the author’s cocitation analysis, we found that Padhye was the most cited, with a total of 146 citations. This shows that Padhye plays an important role in promoting and developing this field. Other authors cited more than 100 times include Sandur SK (138), Checker R (118), Aziz MH (117), Murashige T (115), Kuo PL (115), Hsu YL (108), Wang CCC (102), and Kawiak A (100; Table 1; Fig. 4D).

**Table 1:** The 10 most cited literatures in the study of plumbagin.

Label	Author	Year	Source	Vol	Page	DOI
Perspectives on medicinal properties of plumbagin and its analogs^[31]^	Padhye et al	2012	Med Res Rev	32	1131	10.1002/med.20235
Emerging role of plumbagin: cytotoxic potential and pharmaceutical relevance towards cancer therapy^[32]^	Tripathi et al	2019	Food Chem Toxicol	125	566	10.1016/j.fct.2019.01.018
Plumbagin inhibits tumorigenesis and angiogenesis of ovarian cancer cells in vivo^[33]^	Sinha et al	2013	Int J Cancer	132	1201	10.1002/ijc.27724
Plumbagin induces cell cycle arrest and apoptosis through reactive oxygen species/c-Jun N-terminal kinase pathways in human melanoma A375.S2 cells^[8]^	Wang et al	2008	Cancer Lett	259	82	10.1016/j.canlet.2007.10.005
Plumbagin inhibits the proliferation and survival of esophageal cancer cells by blocking STAT3-PLK1-AKT signaling^[34]^	Cao et al	2018	Cell Death Dis	9	0	10.1038/s41419-017-0068-6
Plumbagin (5-hydroxy-2-methyl-1,4-naphthoquinone) suppresses NF-kappaB activation and NF-kappaB-regulated gene products through modulation of p65 and IkappaBalpha kinase activation, leading to potentiation of apoptosis induced by cytokine and chemotherapeutic agents^[17]^	Sandur et al	2006	J Biol Chem	281	17023	10.1074/jbc.M601595200
Plumbagin, a medicinal plant-derived naphthoquinone, is a novel inhibitor of the growth and invasion of hormone-refractory prostate cancer^[35]^	Aziz et al	2008	Cancer Res	68	9024	10.1158/0008-5472.CAN-08-2494
Plumbagin induces ROS-mediated apoptosis in human promyelocytic leukemia cells in vivo^[36]^	Xu and Lu	2010	Leukemia Res	34	658	10.1016/j.leukres.2009.08.017
Plumbagin-induced apoptosis in human prostate cancer cells is associated with modulation of cellular redox status and generation of reactive oxygen species^[37]^	Powolny and Singh	2008	Pharm Res	25	2171	10.1007/s11095-008-9533-3
Anticancer properties and pharmaceutical applications of plumbagin: a review^[38]^	Liu et al	2017	Am J Chinese Med	45	423	10.1142/S0192415X17500264
Plumbagin, a medicinal plant (Plumbago zeylanica)-derived 1,4-naphthoquinone, inhibits growth and metastasis of human prostate cancer PC-3M-luciferase cells in an orthotopic xenograft mouse model^[39]^	Hafeez et al	2013	Mol Oncol	7	428	10.1016/j.molonc.2012.12.001

#### 3.3.2. Keyword analysis

Keywords often encapsulate the main points of an article, aiding researchers in understanding current research hotspots and trends. Therefore, conducting keyword co-occurrence analysis can provide insights into the application progress of PLB in biomedicine. The keyword co-occurrence network reveals the main research hotspots in related fields, allowing for an understanding of specific research directions. Keyword contributions can be clustered based on their similarity to form keyword clusters.

**Figure 5.**
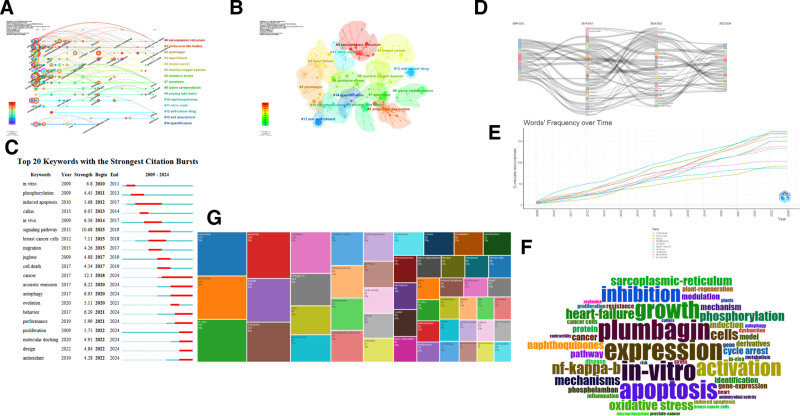
(A) Timeline distribution of cluster analysis of keyword. (B) Cluster analysis. (C) Representative burst keywords. (D) Keyword evolution analysis. (E) Number of active keywords per year. (F) World cloud of key word. (G) The frequency of active keywords.

CiteSpace’s keyword clustering analysis results showed a *Q* value of 0.8859 and an *S* value of 0.932. The top 5 keywords in the cluster are “sarcoplasmic reticulum,” “protocorm like bodies,” “plumbagin,” “heart failure,” and “breast cancer.” CiteSpace illustrates the evolution of keywords in different clusters over time, indicating that the focus of PLB research has evolved from “juglone” to “protein kinase a” (Fig. 5A, B).

Emerging academic trends and new themes are revealed through keyword bursts, which can predict cutting-edge research directions and potential hotspots in specific research fields. The red line indicates the duration of explosive words. The explosive words with the highest intensity are “cancer” (strength = 12.3), followed by “signaling pathway” (strength = 10.68) and “acoustic emission” (strength = 8.22). The longest duration is also “cancer” (2018-2024, 6 years), indicating significant research potential in anti-cancer treatment. From 2022 to the present, the current research frontiers include “antioxidant,” “design,” “molecular docking,” and “promotion,” providing researchers with relevant research directions (Fig. 5C).

Analyzing the evolution of keywords related to PLB research over the past 15 years, we found that the regulatory effect of PLB on biological activity and its mediation of apoptosis in cancer cells have been hotspots (Fig. 5D).

Additionally, using Bibliometrix, we analyzed key words from the past 15 years. “Expression,” “plumbagin,” “in vitro,” “apoptosis,” and “growth” emerged as prominent keywords. This further indicates that PLB research predominantly focuses on cell apoptosis and growth, primarily conducted in vitro, suggesting the need for further exploration to advance it for clinical treatment. We also noticed an increased occurrence of “NF-κB,” indicating its significance in PLB-related research (Fig. 5E–G).

## 4. Discussion

In recent years, the research of PLB related fields has attracted more and more attention, and there are more and more publications. This study quantitatively analyzed 2061 publications on PLB research in the WOS database from 2009 to 2024. The visualization tools CiteSpace and bibliometric were used to analyze the main knowledge areas and emerging trends involved in PLB. Using bibliometric methods, this study quantitatively analyzed the national institutions, published journals, authors, international cooperation and key words in the field of PLB, in order to understand the latest progress, evolution path, frontier research hotspots and future research trends in this field. Since 2009, the number of literatures related to PLB has been increasing, indicating that this field has broad research prospects. At present, this research field is in a stable development stage. These results indicate that the research of PLB is increasingly globalized. In terms of countries and regions, China, the United States and India are the countries with the largest number of documents, and the 3 countries cooperate closely. From the perspective of geographical future, the geographical locations involved in the research of PLB are mainly Asia and North America. From the point of view of the issuing institutions, institutions such as the Institute of Materia Medica and the Academy of plant sciences showed high enthusiasm for the research of PLB. Strengthening the cooperation between the Institute of medicine and the Institute of Biochemistry can better expand the application prospect of PLB. Through bibliometric analysis of published journals, we found out the core journals. Journal of biological chemistry is the most cited journal, and studies related to PLB are widely welcomed in the Journal (if = 4.238, *Q*2). Nature is the most influential journal (if = 64.8, *Q*1), which shows that PLB is worthy of in-depth study and has great promise for future disease treatment. In this study, it was found that almost all of the top 10 cited articles were research on the disease treatment of PLB, and most of them focused on Oncology (n = 6). Most of these studies focused on the biological behavior of cancer cells, regulation of reactive oxygen species, and chemosensitivity of PLB, which shows that PLB shows strong anti-cancer ability and is expected to be used in future clinical anti-cancer treatment. The author of PLB with the largest number of publications is Thomas, David D. His research mainly focuses on the field of plant science and Ca^2+^. Through the co citation analysis of the authors, we found that Padhye was the most cited author, which showed his authority in the research field of PLB. His research on PLB mainly focused on medicinal chemistry and biological activities, and a unified review of the antioxidant, anti-inflammatory, anticancer, antibacterial and antifungal activities of PLB. Keyword cluster analysis showed that the related studies of PLB focused on arcoplasmic reticulum, protocol like bodies, PLB, heart failure, breast cancer. Arcoplasmic reticulum mainly involves inflammation, apoptosis and regulation of reactive oxygen species. Protocol like bodies are mainly involved in plant mass propagation and breeding. Research on heart failure and PLB mainly focuses on the inhibitory effect of PLB on nox-4 activity. The research of PLB on breast cancer mainly focuses on the relationship between pi3k/akt/mtor and NF-κB pathway regulation. Pi3k/akt/mtor pathway is mainly involved in the regulation of cell proliferation, growth, metabolism, angiogenesis and metastasis.^[31,32]^ In recent years, this pathway is considered to promote the success of cancer targeted therapy in the future.^[33,34]^ NF-κB pathway is based on NF-κ. The role of B in the expression of pro-inflammatory genes, including cytokines, chemokines, and adhesion molecules, has been considered as a prototypical pro-inflammatory signaling pathway.^[35,36]^ NF-κ the B transcription factor family plays important roles in cell differentiation and proliferation, immune response, inflammation, and cancer.^[37,38]^ NF-κB as one of the nodal pathways, is related to the occurrence and development of many diseases, such as cancer, inflammation and autoimmune diseases, which further illustrates the wide research scope and wide application prospect of PLB.^[39]^ These results indicate that PLB is expected to be used in the treatment of inflammation and cancer in the future.

Although the bibliometric analysis provided in this paper is innovative, our study still has some limitations: although WOS is the world’s most comprehensive information resource collection database,^[8]^ it does not contain all the research literature of PLB. Only English literature was included in this study, and a small number of literature in other languages may have been omitted. Due to the lag of publication time, some newly published articles have not yet been included in the analysis and attracted attention. The bibliometric software used for analysis also has some limitations. The software can only analyze the information such as the sending institution, the sending author, and the keywords, and lacks the analysis of the specific content in the included research articles.

## 5. Conclusion

This study represents the first endeavor to visualize and analyze research articles on PLB using CiteSpace and bibliometry. It identifies significant countries, journals, and authors involved in PLB-related research and analyzes emerging frontiers in this field, shedding light on current trends. The utilization of PLB in cancer treatment stands out as a primary research focus, warranting further investigation into its specific anticancer mechanisms and modes of action. Meanwhile, with the proposal of the concept of tumor microenvironment, PLB may be one of the potential drugs to remodel the tumor immune microenvironment in the future, which may be a direction worthy of research. Overall, PLB holds considerable research potential. Through objective visualization and analysis, this study offers a bibliometric reference for stakeholders in the field.

## Author contributions

**Data curation:** Zichen Xu, Xinjian Zhang, Jianwei Liu, Runying Guo.

**Formal analysis:** Zichen Xu, Yuqi Xin, Qingkun Jiang.

**Investigation:** Yang Liu.

**Writing – original draft:** Zichen Xu, yang Liu, Jiaxuan Qiu.

**Writing – review & editing:** Yang Liu, Jiaxuan Qiu.
